# A loophole in soap dispensers mediates contamination with Gram‐negative bacteria

**DOI:** 10.1002/mbo3.1384

**Published:** 2023-09-29

**Authors:** Ralf Lucassen, Nicole van Leuven, Dirk Bockmühl

**Affiliations:** ^1^ Faculty of Life Sciences Rhine Waal University of Applied Sciences Kleve Germany

**Keywords:** biofilm, contamination, liquid soap, *Pluralibacter gergoviae*, *Pseudomonas aeruginosa*, pump dispenser

## Abstract

Liquid soap dispensers are widely used in domestic and clinical settings. In previous studies, the risk of bacterial contamination of refillable systems was pointed out and a bacterial contamination rate of 25%, with values of up to 10^8^ colony‐forming units/mL (CFU/mL), was reported. However, the route of contamination remains elusive. To address this point, we determined the microbial contamination of refillable standard pump dispensers and nonrefillable press‐dispenser systems. Following the collection of 104 liquid soap dispensers from hotel rooms across Germany, bacterial counts were determined. Isolates of samples containing nonfastidious Gram‐negative^(lac−)^ bacteria were further analyzed by the Vitek 2 system for the determination of species. 70.2% of the refillable pump dispensers (mean total bacterial count = 2.2 × 10^5^ CFU/mL) but only 10.6% of the nonrefillable press dispensers, were contaminated (mean total bacterial count = 1.5 × 10^1^ CFU/mL). Of samples containing *nonfastidious Gram‐negative* ^(lac−)^ bacteria, *Pluralibacter gergoviae* was present in 41.7%, *Pseudomonads* (*Pseudomonas aeruginosa* and *Pseudomonas putida*) in 25%, *Serratia marcescens* in 16.7%, and *Klebsiella oxytoca* and *Pasteurella testudinis* in 8.3%. After the initial assessment, we contaminated different dispensing systems with *P. aeruginosa/P. gergoviae*, to reveal the route of contamination and identied the pressure release of standard pump dispensers as the loophole for microbial contamination.

## Graphical abstract

Liquid soap dispensers are widely used in domestic and clinical settings. In previous studies, the risk of bacterial contamination of refillable systems was pointed out and a bacterial contamination rate of 25%, with values of up to 10^8^ colony‐forming units/mL was reported. We contaminated different dispensing systems with *Pseudomonas aeruginosa* /*Pluralibacter gergoviae* biofilm, to reveal the route of contamination and identified the pressure release of standard pump dispensers as the loophole for microbial contamination.

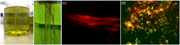

## 1 INTRODUCTION

Hand hygiene is a simple yet very important practice to reduce transmission of infection in healthcare settings. This rule also applies to public and domestic areas and the importance of proper hand hygiene in the food sector is emphasized (Todd et al., 2010; Toney‐Butler et al., 2022). It was furthermore reported that handwashing promotion at child day‐care facilities or schools, can prevent around one‐third of diarrhea cases in high‐income countries and that the risk of *influenza A* infection can readily be reduced by good hygiene habits, especially frequent handwashing with soap (Ejemot‐Nwadiaro et al., 2015; Liu et al., 2016). For this reason, institutions like the World Health Organization (WHO) promote hand hygiene as a preventive measure in all One Health areas (WHO, 2023).

However, the positive effect of handwashing could be mitigated by using liquid soap from bulk‐soap refillable dispensers, contaminated with microorganisms. This is also supported by Zapka et al. (2011), who found that handwashing with contaminated soap from bulk‐soap refillable dispensers, can increase the number of opportunistic pathogens on hands and may play a role in the transmission of bacteria in public settings. In their study, they revealed that one out of four dispensers in public restrooms contains Gram‐negative bacteria, ranging from 10^3^ to 10^8^ colony‐forming units/mL (CFU/mL). To determine the effect of handwashing with contaminated liquid soap, they experimentally contaminated liquid soap with a high level (3.2 × 10^7^ CFU/mL) or low level (3.2 × 10^4^ CFU/mL) of *Serratia marcescens* and found postwash titers of 1.9 × 10^5^ and 5.0 × 10^1^ CFU/hand, respectively. They further confirmed these results in a follow‐up field study. Here the mean number (3.9 × 10^2^ CFU/hand) of bacteria recovered from the hands, after handwashing with a contaminated soap, was significantly higher than the pre‐handwashing value of 1.5 × 10^1^ CFU/hand (Zapka et al., 2011). Another study by Buffet‐Bataillon et al. (2009) found that soap dispensers can act as a continuous source of *S. marcescens*, facilitating handborne transmission of *S. marcescens* by healthcare workers. Furthermore, Lanini et al. (2011) found a link between infected patients and a liquid soap dispenser as a common continuous source of *Pseudomonas aeruginosa*. However, Blanc et al. (2016) revealed in another study, that only 4% of *P. aeruginosainfected* patients carried the original strain from a contaminated liquid soap dispenser (Blanc et al., 2016; Lanini et al., 2011). In any case, the route of microbial contamination of the dispensers themselves remains elusive.

Noteworthy, according to DIN EN ISO 17516: 2015 ‐ 2 and regulations of the European Union the acceptable limit for total aerobic mesophilic bacteria is ≤10^3^ CFU/mL or CFU/g, for cosmetic products of Category 2, which applies to liquid soap dispensers. For *Escherichia coli, P. aeruginosa, Staphylococcus aureus*, and *Candida albicans* this limit is zero. A higher number of total aerobic mesophilic bacteria in a cosmetic product of Category 2, should lead to a locking of products from the ongoing production and shipment (International Organization for Standardization, 2014; Scientific Committee on Consumer Safety, 2022; Neza and Centini, 2016). Our study was conducted to further investigate the extent of contamination of liquid soap dispensers in a public setting and to accurately determine the pathway of contamination. In this respect, we conducted a two‐way study.

## 2 MATERIALS AND METHODS

### 2.1 Collection of liquid soap dispensers from hotels

**Figure 1:**
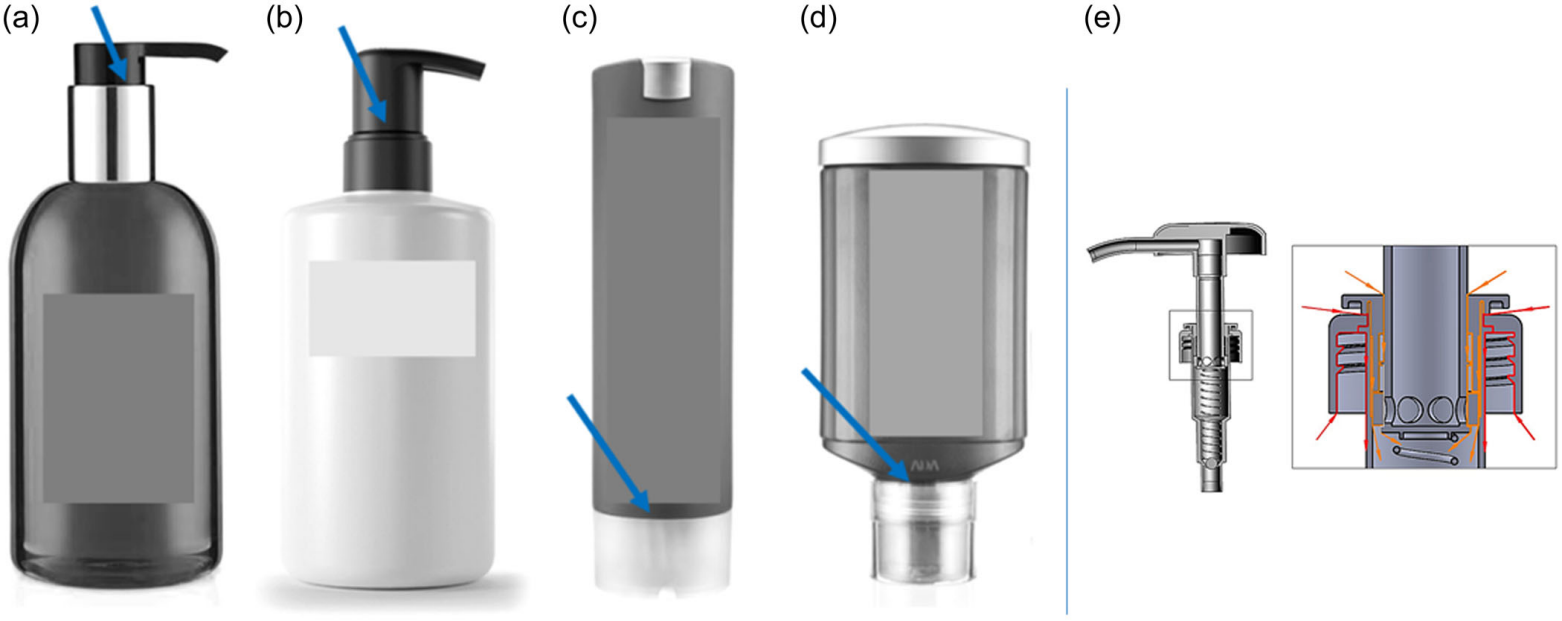
From left to right, refillable standard pump‐dispenser without drainage of the pump head (a), standard pump‐dispenser with drainage of the pump head (b), press‐dispenser type 1 (c), press‐dispenser type 2 (d). Blue arrows indicate the application zone of 200 µL biofilm suspension and tap water (plus 0.1% TSB) respectively. (e) 3D drawing of a standard pump‐dispenser pump head without a drainage system. Stagnant liquid in the pump head leads to biofilm formation. These biofilms are aspirated into the liquid soap via the pressure release when the pump is actuated. Red arrows indicate strong, and orange arrows, a medium influx of liquid/biofilm when the pump is actuated AutoCAD 21.0 Technical Drawing was provided by J. Hans (Faculty of Technology and Bionics of Rhine Waal University). 3D, three‐dimensional; TSB, tryptic soy broth.

A total of 57 standard liquid soap pump dispensers (refillable) and 47 alternative press dispensers (nonrefillable) were collected from hotel rooms across Germany (Figure 1a–d). The dispensers were collected when the liquid soap level was between 75% and 25%. Liquid soap samples were drawn not later than 24 h after collection.

Because liquid soap dispensers are touched daily with dirty hands, the exterior can be heavily contaminated. Thus, the dispenser nozzle was wiped down with a sterile cloth to remove superficial contamination before sampling. The liquid soap from the first three pump/press steps was discarded, to subsequently draw the liquid soap for further analysis.

### 2.2 Cell counts and bacterial species identification by Vitek 2 system (Biomerieux)

Because of the viscous nature of liquid soap, direct application of the sample to agar plates and determination of CFU/mL is not possible. Liquid soap samples were therefore diluted from 10^−1^ to 10^−4^ in neutralizing solution (10 mM phosphate‐buffer saline (PBS), 3% Tween 80, 0.3% lecithin, 0.1% histidine, and 0.01% thiosulfate) and spread‐plated by application of 100 µL to tryptic soy agar (TSA), malt extract agar, or MacConkey agar (Merck), for determination of total bacterial count, yeasts/molds, and nonfastidious Gram‐negatives, respectively. Plates were incubated for 24–48 h at 30°C. The lowest dilution of 10^−1^ also means that samples with CFU/mL values below 100 must be considered as ≤100 CFU/mL. Triplicate determinations were carried out.

Nonfastidious Gram‐negative^(lac−)^ colonies (MacConkey agar) of each sample were classified based on colony morphology (light pink, pale, or colorless colonies either mucoid, flat smooth, flat jagged, or convex smooth).

Of these groups, isolates were picked and subcultured on TSA. After sub‐cultivation for 24–48 h at 30°C, pure isolates were applied to the VITEK 2 System/VITEK 2 GN ID card (Biomerieux) for the determination of bacterial species.

We focused on this group as it contains many critical priority bacteria (defined by WHO) such as *Acinetobacter baumannii*, *P. aeruginosa*, and some Enterobacteriaceae (e.g., *Pluralibacter gergoviae*, *Stenotrophomonas maltophilia*, *S. marcescens*, *Citrobacter* spp.). These pathogens exhibit intrinsic resistance to multiple antibiotics and can cause severe and often fatal infectious diseases such as bloodstream infections and pneumonia. Furthermore, some of these strains can use liquid soap ingredients as substrates and exhibit increased tolerance to common preservatives (Ambily & Jisha, 2014; Cheng & Chen, 1994; Périamé et al., 2014; Weiser et al., 2019).

*P. aeruginosa* isolate of sample 42 and *P. gergoviae* isolate of sample 26, were used for the in vitro/in situ experiments.

### 2.3 Preparation of bacterial suspension for in situ experiments

*P. aeruginosa* and *P. gergoviae* were incubated in 10% tryptic soy broth (TSB) at 30°C for 48 h. These cultures contain free planktonic cells, but also bacterial aggregates embedded in an extracellular matrix with biofilm properties. A 1/1 mixture of *P. aeruginosa* and *P. gergoviae* culture in tap water was prepared (total bacterial count of 2.5 × 10^5^ CFU/mL). Concentration was determined by spread plating on TSA after vigorous vortexing for 1 min.

### 2.4 In situ assessment of liquid soap dispensing systems for their safety against microbial contamination

During the second phase of this study, it became clear that the main route of microbial contamination is the pressure release of the pump dispensers. Liquid can accumulate and is aspirated into the pump head and further into the dispenser bottle through the pressure release when the pump is actuated. Based on this observation we developed the following sequence for a contamination model. Application of 200 µL bacterial suspension (see Section 2.3) was followed by continuous application of 200 µL of sterile tap water plus 0.1% TSB and dispensing every second day with three pump strokes, over a period of 40 days. We used these two species because they were found in high numbers in our and other studies in liquid soaps and further cosmetics as well (Neza & Centini, 2016). This sequence takes into consideration that only small volumes of liquid enter the pump head and that it contains low amounts of nutrients, from e.g. dirty hands. However, the frequency and type of use of dispensers are very diverse and cannot be fully simulated.

The following dispenser types were tested in triplicate determination (see Figure 1). Dispensers A and B are standard dispensers. Here, the liquid that enters the pump head cannot drain off and remains in the pump head. Press‐dispensers C and D, on the other hand, allow liquids that enter the head to drain off and no stagnant liquid can thus accumulate. Figure 1e depicts the pump head mechanism of standard pump dispenser A (without a drainage system). Determination of viable counts was carried out according to Section 2.2 on TSA plates.

### 2.5 In vitro assessment of bacterial growth in liquid soap

To assess whether the most prominent isolates, found in liquid soap pump dispensers from hotels (*P. aeruginosa* and *P. gergoviae*), can grow in standard liquid soap, we conducted a series of experiments. Liquid soap (freshly opened; formulation see Table 1) was diluted in sterile tap water (6.8°dH) or 10% TSB to a concentration of 12.5%, 25%, 50%, and 75% in a final volume of 25 mL (wt/vol) in 50 mL test tubes. This way the soap concentration decreases while nutrient concentration increases. Dilutions and the nondiluted liquid soap (100%) were inoculated with 1 mL of bacterial suspension and mixed by end‐over‐end rotation until a homogenous suspension was reached. Incubation was done at 21°C over 28 days. Triplicate determinations were carried out. At time point 0 and then weekly, 1 mL of the sample was drawn by a micropipette. Determination of viable counts was done according to section 2.2 on TSA plates.

**Table 1:** Formulation of standard liquid soap.

Ingredients		%
Aqua		80–85
Sodium Laureth Sulfate		8–13
Cocoamidopropyl Betaine		2–5
Sodium Chloride		1–3
Glycerin		≤0.5
Lactic Acid		≤0.1
Tocopherol		≤0.001
Coco‐glucoside		≤0.5
Glyceryl Oleate		≤0.5
Propylene Glycol		≤0.5
Hydrogenated Palm Glycerides		≤0.0001
Citric Acid		0.1–0.5
Benzophenone‐1		≤0.5
Sodium Sulfate		≤0.001
Potassium Hydroxide		≤0.00001
Sodium Benzoate		0.1–0.2
Potassium Sorbate		0.05–0.15
Fragrance/Color		0.5–1.0
Leaf Extracts		≤0.1

### 2.6 Analysis of biofilms by fluorescence microscopy

Biofilm samples were collected by swab method from surfaces or by simple aspiration, using a micropipette. The biofilm was stained using Filmtracer™ LIVE/DEAD™ Biofilm Viability Kit (Invitrogen).

The biofilm was transferred to a microscope glass slide and submerged in 250 µL of a staining solution (1 μL of SYTO® 9 stain and 1 μL of propidium iodide stain per 1 mL of 10 mM PBS pH 7.2). The glass slide was covered using a plastic Petri dish lid and incubated and protected from light (20 min at room temperature). After incubation, the staining solution was aspirated, leaving the biofilm attached to the surface of the glass slide. To remove access dye, the biofilm was rinsed with 500 µL of 10 mM PBS pH 7.2. After the staining procedure, the biofilms were analyzed using a Zeiss Axio Imager M2M Fluorescence Microscope and a water immersion objective (Zeiss, Oberkochen, Germany).

### 2.7 In situ staining of biofilms in liquid soap

In situ staining of biofilms in liquid soap was carried out by application of 200 µL of resazurin solution (6 mg/mL in deionized distilled H_2_O; Sigma Aldrich, St. Louise, USA) directly to the pump head, followed by three pump strokes. After 24–48 h the staining was complete.

### 2.8 Statistical methods

We used GraphPad Prism (Version 6.07) for statistical calculations. Determination of the statistical significance of the respective cell numbers between refillable pump‐ and nonrefillable press‐dispensers and the significance of the growth of bacteria in different concentrations of liquid soap was done by Dunn's multiple comparison test and Tukey's multiple comparison test, respectively.

## 3 RESULTS

### 3.1 Determination of microbial contamination of liquid soap dispenser systems from hotel rooms across Germany

**Figure 2:**
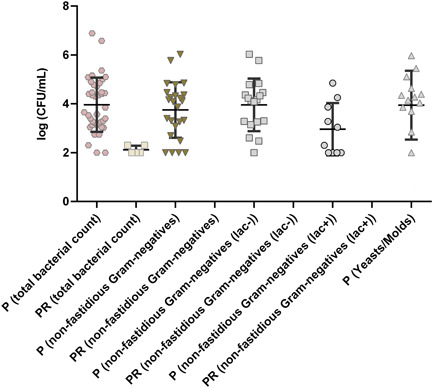
Mean values and standard deviation of viable cell counts (CFU/mL) of refillable standard pump‐dispensers (P) and nonrefillable press‐dispensers (PR), collected from hotels across Germany. Asterisks indicate the significance level based on *p*‐values (*p = 0.05, **p = 0.01, ***p = 0.001, ****p = 0.0001, ns = not significant) between pump‐ and press‐dispensers. CFU, colony‐forming unit.

Figure 2 gives an overview of the mean viable cell counts between the refillable standard pump‐ (P) and nonrefillable press‐dispensers (PR). The standard pump dispensers had no drainage of the pump head.

Except for *nonfastidious Gram‐negatives* ^(lac+)^, the difference in mean values (CFU/mL) between the pump‐ and press‐dispensers is significant (see Figure 2 legend for *p*‐values).

Of 57 standard pump dispensers, 70.2% (40/57) were contaminated with bacteria (mean total bacterial count = 2.2 × 10^5^ CFU/mL; max. 7.7 × 10^6^ CFU/mL). 31.6% of the pump dispensers (18/57) contained *nonfastidious Gram‐negative* ^(lac−)^ bacteria (mean total viable count = 3.3 × 10^4^ CFU/mL; max. 1.1 × 10^6^ CFU/mL). Yeasts and molds were found in 13/57 of the pump dispensers (22.8%; mean cell count = 2.6 × 10^4^ CFU/mL; max. 9.4 × 10^5^ CFU/mL).

In contrast to the results of the pump dispensers, the press dispensers exhibited a very low contamination rate of 10.6% (5/47) and only very low cell counts (mean total bacterial count = 1.5 × 10^1^ CFU/mL; max. 1 × 10^2^ CFU/mL) and neither *nonfastidious Gram‐negatives* nor yeasts and molds were detected. The mean total viable counts were therefore close to the detection limit of the analytics used here, of ≤100 CFU/mL.

### 3.2 Bacterial isolates of dispensers collected from hotels across Germany

Individual bacterial species were isolated from plates with *nonfastidious Gram‐negative* ^(lac−)^ *colonies*. This pertains only to the standard pump dispensers, as shown in Figure 2. Bacterial isolates could be subcultured from 12 of 18 samples. *P. gergoviae* was detected in 5/12 (41.7%), pseudomonads (*P. aeruginosa* and *Pseudomonas putida*) in 3/12 (25%), *S. marcescens* in 2/12 (16.7%), and *Klebsiella oxytoca* and *Pasteurella testudinis*, respectively, in 1/12 (8.3%) of the tested samples.

Interestingly, if liquid soaps from hotels were heavily contaminated, one or two bacterial species dominated.

### 3.3 Growth of *P. aeruginosa* and *P. gergoviae* in diluted and nondiluted liquid soap

**Figure 3:**
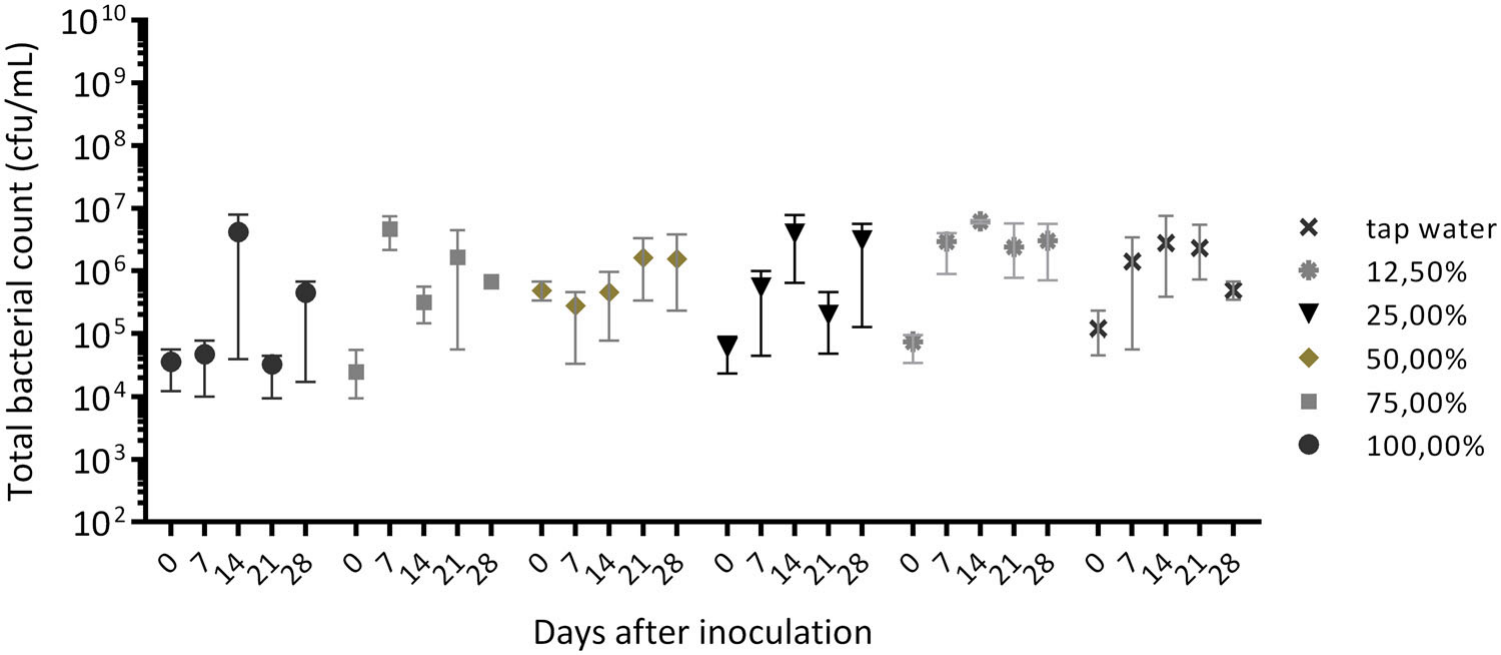
Mean total bacterial counts with standard deviation (*Pluralibacter gergoviae* and *Pseudomonas aeruginosa*) in descending concentrations of liquid soap solution diluted in tap water.

**Figure 4:**
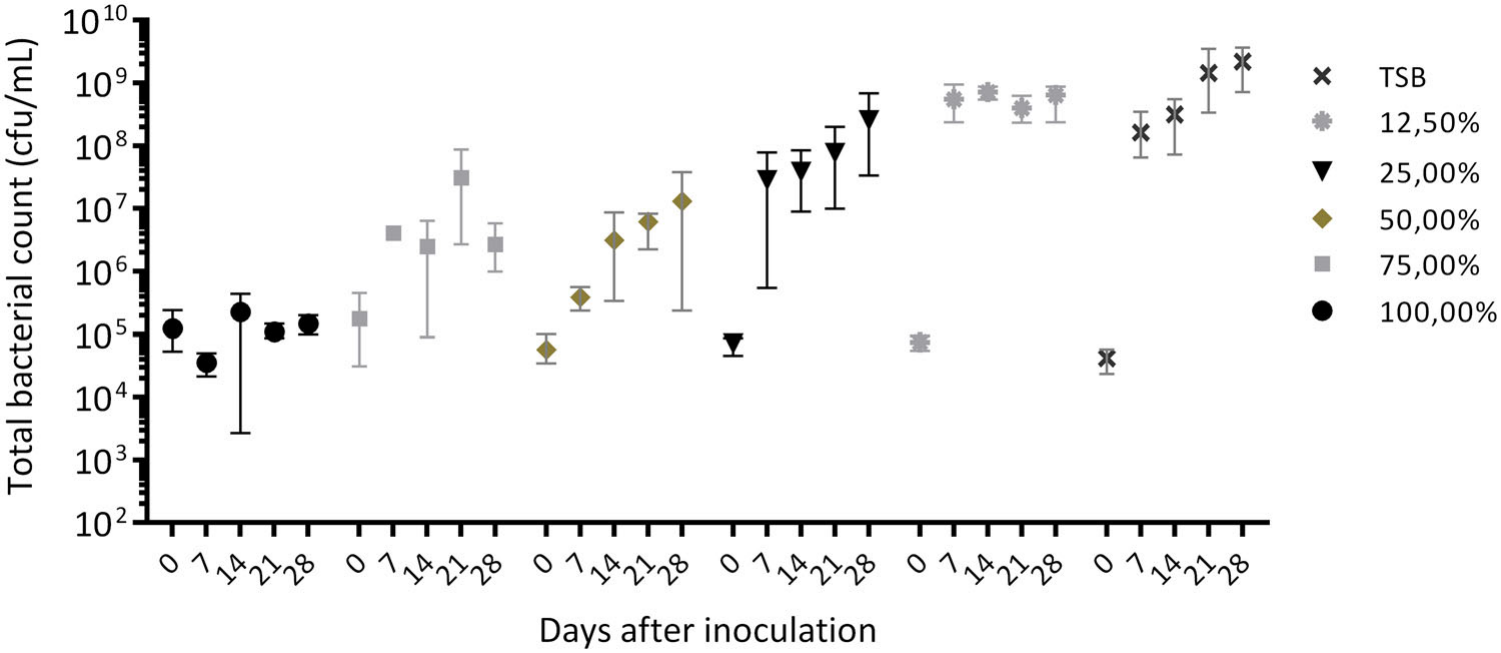
Mean total bacterial counts with standard deviation (*Pluralibacter gergoviae* and *Pseudomonas aeruginosa*) in descending concentrations of liquid soap solution diluted in 10% TSB. TSB, tryptic soy broth.

In 100% liquid soap solution, no growth of bacteria was observed, and the bacterial count remained at the level of Day 0 (Figures 3 and 4). The increase and decrease of cell numbers are not significant, as determined by Tukey's multiple comparison test. This is a result of the viscous nature of the sample matrix and the inhomogeneous distribution of the cells/cell aggregates, which made reproducible sampling difficult.

When diluted in tap water, slight growth occurs with decreasing soap concentration but only by 1–2 log10 steps (Figure 3). Although this slight increase is not significant, we assume that some growth occurs due to the residual nutrients from the inoculum (*P. gergoviae* and *P. aeruginosa* bacterial suspension in 10% TSB) but that the bacteria cannot use the soap solution as a substrate alone. In any case, significant growth in 100% liquid soap solution was not present. In addition, the liquid soap solution and its preservation system are likely to have an inhibitory effect. However, it is important to note that we found no decrease in cell number in 100% liquid soap over 28 days.

**Figure 5:**
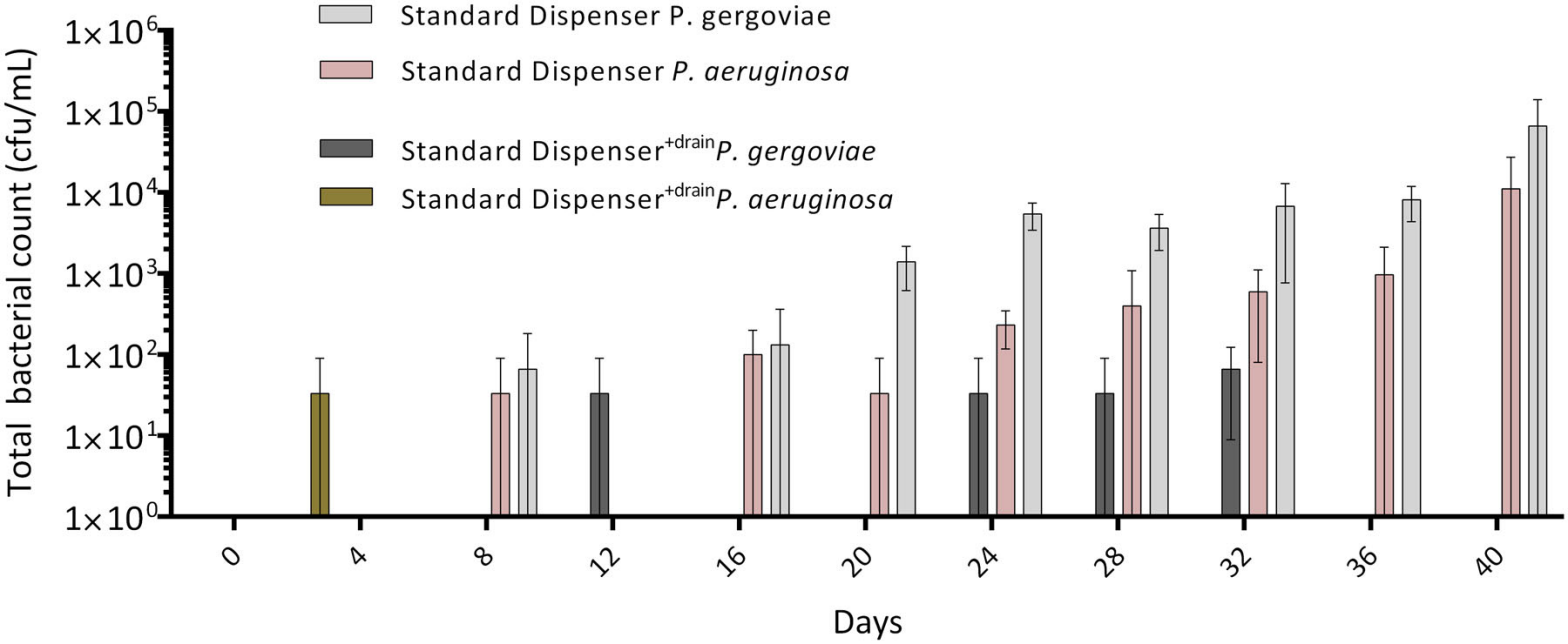
Mean total bacterial counts with a standard deviation of liquid soap from standard pump dispensers, with and without a pump‐head drainage system. Dispensers were inoculated on Day 0 with 200 µL of a *Pseudomonas aeruginosa/Pluralibacter gergoviae* biofilm suspension, followed by application of 200 µL sterile tap water (0.1% TSB) to the pump head every second day and actuation of the pump. TSB, tryptic soy broth.

Diluted in 10% TSB, as the soap concentration decreases and the nutrient level increases, significant growth occurred by up to 3–4 log10 steps at 12.5%–75% liquid soap (Figure 5). This means, that as soon as a certain level of nutrients is available and the soap concentration is lower, the bacteria can proliferate. This is particularly the case when an aqueous phase settles on top of the liquid soap and forms a zone of diluted soap at the air–liquid interface. Especially at 75% liquid soap, a very strong biofilm formation occurred, with the biofilm floating on the soap phase.

It must also be emphasized that we were not able to adapt the mixed biofilm to 100% liquid soap, which means that even *P. aeruginosa* and *P. gergoviae*, which were adapted to 50%–75% liquid soap, were not able to proliferate in undiluted liquid soap within the incubation period of 28 days.

### 3.4 Evaluation of liquid soap dispenser systems regarding their microbial safety in our contamination model

We tested two types of pump and press liquid soap dispensing systems (Figure 1) in our contamination model.

**Figure 6:**
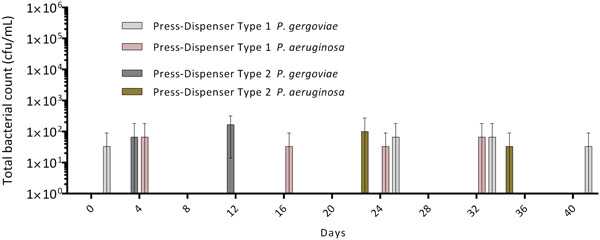
Mean total bacterial counts with standard deviation of liquid soap press‐dispensers, inoculated on Day 0 with 200 µL of *Pseudomonas aeruginosa/Pluralibacter gergoviae* biofilm suspension, followed by application of 200 µL sterile tap water (0.1% TSB) every second day and actuation of the press‐dispenser. TSB, tryptic soy broth.

After contamination with *P. aeruginosa/P. gergoviae* bacterial suspension (see Section 2.3) and continuous application of 200 µL of sterile tap water (plus 0.1% TSB) and actuation every second day, over the period of 40 days, we found increasing total bacterial counts in the standard pump dispensers without a pump head drainage system. In contrast, only some standard pump dispensers with a pump head drainage showed low contamination close to the detection limit of ≤100 CFU/mL (Figure 5) and no increase over time. In the press‐dispenser systems, very low mean CFU/mL were found, at concentrations close to the detection limit (Figure 6). Here also, no increase of CFU/mL throughout the experiment was observed. We thus concluded that these bacteria originated from the outer part of the dispensers.

### 3.5 Analysis of biofilms

Biofilms were present not only on all standard pump dispensers and here especially on and in the pump head, but also in cavities of other dispenser types that contained stagnant water.

**Figure 7:**
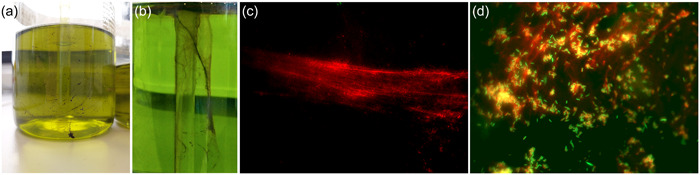
(a) Biofilm in a standard pump dispenser collected from a hotel in Germany. (b) Biofilm along the riser tube of an artificially contaminated standard pump dispenser, stained with *Resazurin*. (c) ×100 microscopic fluorescent image of *Pluralibacter gergoviae/Pseudomonas aeruginosa* biofilm matrix, isolated from a liquid soap pump‐dispenser after 40 days in our contamination model (stained with *propidium iodine*). (d) ×1000 microscopic fluorescent image of *P gergoviae/P aeruginosa* biofilm isolated from a liquid soap pump‐dispenser after 40 days in our contamination model (live/dead stain with propidium iodide and SYTO9).

Figure 7b–d depicts a biofilm of a liquid soap pump‐dispenser that was artificially contaminated in our contamination model. Noteworthy, we found the same filamentary mucus‐like structures, we also found in the original dispensers collected from hotels (Figure 7a).

The biofilm matrix was extremely slimy and formed strong filamentous structures. It contained a large amount of extracellular DNA (eDNA) as can be seen by propidium iodine staining (Tang et al., 2013) in Figure 7c. Figure 7d shows that most bacterial cells in close proximity to filamentous eDNA were dead or less vital (red fluorescence by propidium iodine). However, a recent study found that propidium iodine staining may underestimate the viability of bacterial cells, due to large amounts of eDNA (Rosenberg et al., 2019). From Figure 5, we conclude that most of the bacteria (90%) in those biofilms were *P. gergoviae*.

## 4 DISCUSSION

70.2% of standard refillable pump dispensers collected from hotel rooms across Germany were contaminated with bacterial biofilms but only 10.6% of the nonrefillable press dispensers. Furthermore, mean CFU/mL values were significantly lower in the press dispensers and close to the detection limit. 18/57 (31.6%) of pump‐dispensers were contained with *nonfastidious Gram‐negative* ^(lac−)^ bacteria. For 12/18 of samples containing *nonfastidious Gram‐negative* ^(lac−)^ colonies, bacterial isolates could be picked and subcultured. *P. gergoviae* was found to be the most frequent (5/12) *nonfastidious Gram‐negative* ^(lac−)^ colonizer. Further isolates included the species *P. aeruginosa*, *P. putida, S. marcescens, K. oxytoca*, and *P. testudinis*.

The species and prevalence in our study are comparable to other studies that have also found a high contamination rate of liquid soap pump dispensers with nonfastidious Gram‐negative^(lac−)^ bacteria (Blanc et al., 2016; Buffet‐Bataillon et al., 2009; Lanini et al., 2011; Lompo et al., 2023; Zapka et al., 2011).

Although *Gram‐negative* bacteria were the main microbial contaminants of liquid soap from pump dispensers, the high number of contamination (22.8%) with yeast and molds found in this study should not be overlooked. Especially considering an increase in fungal infections and antifungal resistance of *Candida* spp. and *Aspergillus fumigatus* (Du et al., 2020; WHO, 2022).

We furthermore found that the pump head of standard liquid soap pump dispensers (Figure 1a) acted as the main entry route for bacterial biofilms. Caused by the accumulation of liquid in the pump head and associated biofilm formation, the biofilms entered the pump dispensers directly via the pressure release when the pump was actuated. Noteworthy, this applied to most of the standard market products.

However, the construction of liquid soap pump‐dispenser systems is very diverse, ranging from simple disposable nonrefillable systems to mounted nondisposable refillable systems. Thus comparison of the contamination routes is difficult. Nevertheless, systems without a pump head or a pump head where liquid cannot accumulate (drainage system), were less or not affected by microbial contamination. Liquid soap from nonrefillable press dispensers was scarcely contaminated and contamination rather originated from the transfer of bacteria, from the outer part of the dispenser, during sampling.

We furthermore found that *P. aeruginosa/P. gergoviae* biofilms rather persist and accumulate, than grow in an undiluted (100%) liquid soap solution. These persisting cells have the potential to shift back to a growth phase and become problematic if conditions are more favorable. However, if the liquid soap is diluted by an influx of liquid through the pump head and an aqueous phase forms on top of the liquid soap, these cell aggregates and biofilms can grow slowly especially with the simultaneous introduction of nutrients. This is also the case when the liquid soap contains a preservative system and is supported by other studies as well. *P. aeruginosa* and *P. gergoviae* can develop resistance to common preservatives and antibiotics and can use a wide range of different substrates (fats, oils, surfactants, etc.) for energy production (Ambily & Jisha, 2014; Cheng & Chen, 1994; Périamé et al., 2014; Weiser et al., 2019).

In our study, no significant growth was observed in undiluted liquid soap (see Table 1 for formulation) over 28 days but refillable dispensers are in use for much longer in reality. Further studies are mandatory to clarify this point. Overall, we conclude that bacterial growth does not occur in an undiluted standard liquid soap. We thus further conclude, that pure standard liquid soap is sufficiently protected by the preservation system. However, if a biofilm enters the interior of the dispenser via the pressure release, it can persist as a floating biofilm on or in the liquid soap. As soon as an aqueous phase builds up in the dispenser bottle due to the introduction of liquid, growth of the bacteria can occur in this diluted soap zone at the air–liquid interface. The strength of the growth also depends on additional nutrients introduced with the liquid, which was simulated in our contamination model through the application of tap water plus 0.1% TSB.

In terms of risks and infection, *P. gergoviae*, in particular, has recently been the focus of the Federal Institute for Risk Assessment (Germany) as well as the Food and Drug Administration (FDA) and *P. gergoviae* as well as *P. aeruginosa* were found to be the main cause of microbial contamination of shower gels, shampoos, liquid soaps, and lotions (OPEN AGRAR, 2020; Sutton & Jimenez, 2012).

We also found that especially *P. gergoviae* can form a mucus‐like, filamentary, and extremely sticky biofilm that contains a lot of eDNA. Extracellular DNA is known to facilitate attachment to surfaces in other species (Pakkulnan et al., 2019). These hallmarks make a transfer and new attachment from contaminated to noncontaminated areas likely.

*P. gergoviae* and *P. aeruginosa* are facultative pathogens. Infections of vulnerable individuals have been described. *P. aeruginosa* is known as the leading cause of morbidity and mortality in cystic fibrosis patients and as one of the leading causes of nosocomial infections (Moradali et al., 2017). In addition, a direct relationship between contaminated liquid soap pump dispensers and infection of patients in a clinical setting was established by other studies (Blanc et al., 2016; Buffet‐Bataillon et al., 2009; Lanini et al., 2011). Infections with *P. gergoviae* are less frequent and have been described in respiratory diseases, urinary tract infections, or endophthalmitis (Chen et al., 2009). *P. gergoviae* infection in otherwise healthy individuals occurs very rarely but clinical outbreaks have been reported (Cantón et al., 2002; Ganeswire et al., 2003). Furthermore, the FDA and the European Union assess the risk of infection for both microorganisms as high enough, to designate contamination of cosmetic products with these “objectional” microorganisms as grounds for locking of products for the ongoing production and shipment or recall (OPEN AGRAR, 2020; Scientific Committee on Consumer Safety, 2022).

Based on the findings of this study, we can make the following statements and recommendations with regard to the safety of dispensing systems for personal care products like liquid soaps, shampoos, and lotions against microbial contamination.

Standard pump dispensers for personal care products can become heavily contaminated with microorganisms through aspiration of biofilms via the pressure release of the pump head. The technical drawing in Figure 1e depicts the entrance route via the pressure release.

To prevent this, the pump head must be protected from stagnant liquid and thus from the formation of biofilms. Pump systems where the liquid can drain off, are much less contaminated, as are standard press dispensers. Refillable systems may also become contaminated during refill, since a biofilm can also form on other parts of the dispenser. An example for this is the threaded connection of the pump head. Thus, the pump head of refillable systems should be replaced regularly. If the threaded connection is heavily contaminated, a new dispenser bottle should be used. Finally, yet importantly jugs or funnels used to refill the dispensers should be cleaned and dried regularly, to prevent the growth of Gram‐negative bacteria and further transfer to the dispensers. Ideally, the dispensers should be refilled directly from the bulk container (Table 2).

**Table 2:** Bacterial isolates from dispensers with *nonfastidious Gram‐negative* ^(lac−)^ bacteria between 10^2^ and 10^6^ CFU/mL.

Sample	Dispenser‐ system	Species	Total bacterial count (CFU/mL)	*Nonfastidious Gram‐negatives* ^(lac−)^ (CFU/mL)
11	P	*Pseudomonas putida*	1.70E + 03	3.00E + 02
11	P	*Pasteurella testudinis*	1.70E + 03	3.00E + 02
17	P	*Pluralibacter gergoviae*	3.73E + 03	1.00E + 02
19	P	*Pluralibacter gergoviae*	1.03E + 05	1.30E + 04
23	P	*Pluralibacter gergoviae*	3.80E + 06	5.90E + 05
25	P	*Pluralibacter gergoviae*	1.23E + 05	1.70E + 04
26	P	*Pluralibacter gergoviae*	7.70E + 06	1.07E + 06
27	P	*Klebsiella oxytoca*	5.90E + 04	2.50E + 03
33	P	*Serratia marcescens*	1.40E + 03	9.00E + 02
42	P	*Pseudomonas aeruginosa*	7.20E + 04	6.80E + 04
43	P	*Pseudomonas aeruginosa*	5.20E + 04	6.10E + 04
43	P	*Serratia marcescens*	5.20E + 04	6.10E + 04

Abbreviation: CFU, colony‐forming unit.

## AUTHOR CONTRIBUTIONS

**Ralf Lucassen**: Investigation (equal); methodology (equal); writing—original draft (equal). **Nicolevan Leuven**: Investigation (equal). **Dirk Bockmühl**: Project administration (equal); writing—review and editing (equal).

## CONFLICT OF INTEREST STATEMENT

The authors declare no conflict of interest.

## ETHICS STATEMENT

None required.

## Data Availability

All data are provided in full in the results section of this paper.
